# Systematic Underreproduction of Time Is Independent of Judgment Certainty

**DOI:** 10.1155/2016/6890674

**Published:** 2016-01-10

**Authors:** Martin Riemer, Darren Rhodes, Thomas Wolbers

**Affiliations:** ^1^Aging & Cognition Research Group, German Center for Neurodegenerative Diseases (DZNE), 39120 Magdeburg, Germany; ^2^Centre for Computational Neuroscience and Cognitive Robotics, School of Psychology, University of Birmingham, Edgbaston, Birmingham B15 2TT, UK; ^3^Center for Behavioral Brain Sciences, 39118 Magdeburg, Germany

## Abstract

We recently proposed that systematic underreproduction of time is caused by a general judgment bias towards earlier responses, instead of reflecting a genuine misperception of temporal intervals. Here we tested whether this bias can be explained by the uncertainty associated with temporal judgments. We applied transcranial magnetic stimulation (TMS) to inhibit neuronal processes in the right posterior parietal cortex (PPC) and tested its effects on time discrimination and reproduction tasks. The results show increased certainty for discriminative time judgments after PPC inhibition. They suggest that the right PPC plays an inhibitory role for time perception, possibly by mediating the multisensory integration between temporal stimuli and other quantities. Importantly, this increased judgment certainty had no influence on the degree of temporal underreproduction. We conclude that the systematic underreproduction of time is not caused by uncertainty for temporal judgments.

## 1. Introduction

The goal of psychophysics is to describe the relation between physical and psychological realms [1], and, to this end, researchers should possess complete control over the physical stimuli used in their experiments [2]. This important principle is violated for the dimension of time [3]. While most physical qualities can be presented in an ascending and a descending manner (e.g., weights can increase and decrease), perceived time always runs in the same direction. This peculiarity is referred to as the anisotropy of time and it can explain some well-known phenomena in timing research, for example, the underreproduction of temporal intervals [4].

**Figure 1:**
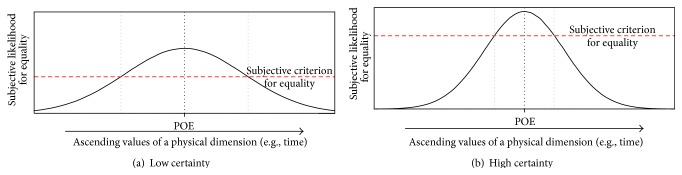
Probability curve for a correct response depending on the elapsed time during the reproduction phase. Increased certainty (b) should result in a sharpening of the curve and in a more restrictive criterion level, thereby attenuating the degree of underreproduction.

In time reproduction, participants are presented with a stimulus of a specific duration (i.e., the standard interval) and afterwards they have to terminate a second stimulus as soon as it has reached the same duration as the standard (i.e., the reproduced interval). Numerous applications of this task have consistently revealed that the reproduced intervals are shorter than the standards [5]. This phenomenon has often been interpreted as an erroneous perception of time, in the sense that the second duration is perceived as longer than the standard, and therefore it is terminated too early (e.g., [6]). This interpretation was questioned in a recent study [4], in which we proposed that the negative errors in time reproduction tasks might also be caused by the asymmetric flow of perceived time. Reproduction tasks are based on the method of limits [2], in which a target value on a specific dimension (e.g., loudness) is approached from smaller or larger values and for which it is pertinent to alter the direction of this dimensional change [3]. In other words, the correct value on a physical continuum (i.e., the presented standard) must be approached alternately from smaller and larger values. However, this important manipulation is not possible with respect to the time dimension. The probability for a correct response changes continuously during the reproduction phase. In the examples depicted in Figure 1, the duration with the highest probability of being judged as equal to the standard would coincide with the point of objective equality (indicating perfect mean accuracy), but all other durations above the criterion level would also cause a termination of the reproduction phase. Due to the anisotropy of perceived time, this criterion level is necessarily reached at smaller values. Taken together, we proposed that the underreproduction of temporal intervals does not occur because shorter durations are more likely to be confounded with the standard than longer durations but rather occurs because shorter durations have to be presented previous to longer ones [3, 4].

One potential cause for the general bias towards earlier responses consists in the uncertainty involved in reproduction tasks. Increasing the certainty of temporal judgments should result in a sharpening of the probability curve for a correct response and in a more restrictive criterion level. As can be inferred from Figure 1, this would give rise to an attenuation of negative reproduction errors. Thus, if the bias towards earlier responses is due to uncertainty, then a reduction of this uncertainty should be accompanied by an attenuation of negative reproduction errors.

A promising method to manipulate uncertainty in timing tasks is provided by transcranial magnetic stimulation (TMS) [7]. Applied over the right posterior parietal cortex (PPC), repetitive TMS selectively influenced the precision in time discrimination tasks, reflecting altered certainty, whilst the mean accuracy of temporal judgments was unaffected [8]. An involvement of the right PPC in the perception of time has also been demonstrated by electrophysiological recordings in monkeys and by functional neuroimaging studies in humans [9–13].

In the present study, we examined judgment certainty as a potential cause for the general bias towards earlier responses in time reproduction and ultimately its role for the systematic underreproduction of temporal intervals. Specifically, we tested whether altered certainty regarding temporal judgments results in an attenuation of negative errors in a time reproduction task. To this end, the right PPC was inhibited via continuous theta-burst stimulation (cTBS; [14–16]). Under TMS and sham stimulation, participants performed a time discrimination and a time reproduction task. Time discrimination data served as a manipulation check, verifying that judgment precision (as an indicator of certainty) was affected by inhibition of the right PPC. Regarding time reproduction, it was hypothesized that the systematic underreproduction of time was attenuated when temporal certainty is high.

## 2. Methods

### 2.1. Participants

Twenty-four participants (9 males, mean age of 26.3 years) were recruited from the local community. All but one were right-handed. Exclusion criteria were metallic objects in the body (due to T1 image acquisition), auditory impairments, or previous occurrences of epileptic seizures. Participants received monetary compensation and gave written informed consent to the experimental protocol, which was approved by the local ethics committee.

### 2.2. Tasks and Stimuli

Participants performed a time discrimination and a time reproduction task (order counterbalanced across participants), each of which lasted about six minutes. In both tasks, durations were signalized by acoustic stimuli (sine wave sounds of 300 Hz) in filled intervals. Durations were in the suprasecond range, because temporal underreproduction has frequently been reported for intervals within this range [5]. Sounds were delivered via noise-cancelling in-ear headphones (Sennheiser CX 300 II) and controlled with Vizard (v4.0).

In the discrimination task, a standard duration of 3 s was presented, and, after an interstimulus interval of 1 s, one of six comparison durations (2.5, 2.7, 2.9, 3.1, 3.3, and 3.5 s). Each comparison was presented five times in a randomized order, accumulating to 30 trials. Participants had to decide in a two-alternative forced-choice task, whether the comparison duration was shorter (button C on a standard keyboard) or longer (button M) than the standard.

In the reproduction task, one of five standard durations (1, 2, 3, 4, and 5 s) was presented, and, after a 1 s ISI, the reproduction interval was started, which was also signalized by a 300 Hz sine wave sound. Participants were instructed to terminate the reproduction interval (button B), once it had reached the same duration as the standard. Participants responded always with their dominant hand. Standards were presented six times each, resulting in 30 trials.

During both tasks, participants were instructed to close their eyes and to refrain from mental counting strategies. Postexperimental interviews revealed no difficulties to adhere to these instructions.

### 2.3. TMS Protocol

The tasks were performed during two experimental sessions. In the TMS session, continuous theta-burst stimulation (cTBS) was applied over the right PPC. In the sham session, the coil was turned upside down and no TMS was applied. Due to this procedure, acoustic disturbance and vibrations of the coil were comparable during TMS and sham sessions. Each session contained either TMS or sham stimulation and was performed on a different day, with the order being counterbalanced across participants.

**Figure 2:**
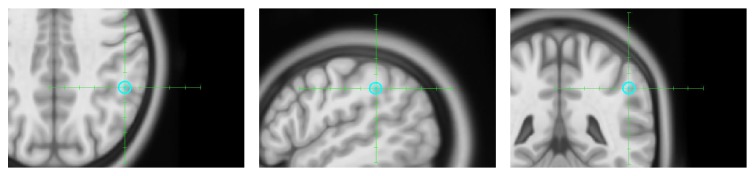
Target stimulation site in the right posterior parietal cortex.

Stimulation site was determined on the basis of individual T1-weighted MRI scans. In each image, we identified the intersection point of Brodmann areas 39, 40, and 7 at the right intraparietal sulcus (IPS; Figure 2). Mean Talairach coordinates across subjects were (51.0, −41.1, 38.8). Navigation of the coil was supported by Localite TMS Navigator (version 2.1.18). As the focus of the present study was not to compare effects between two different stimulation sites but instead to test whether time discrimination and time reproduction tasks are differentially affected by PPC inhibition, we did not include a control stimulation site.

TMS was controlled by a MagPro stimulator (X100+MagOption, MagVenture) and pulses were delivered by a water-cooled figure-of-eight coil with an outer diameter of 75 mm (Cool B-65, MagVenture). We applied continuous theta-burst stimulation (cTBS) according to the protocol described in Nyffeler et al. [16] and in Chaves et al. [14]. Bursts containing three biphasic pulses (repeated at 30 Hz) were applied for 44 s at 6 Hz. Thus, TMS consisted of 267 bursts (801 single pulses). During stimulation, participants wore noise-cancelling in-ear headphones.

Pulse intensity was individually set to 100% of the resting motor threshold (MT), which was defined as the lowest intensity capable of inducing a motor evoked potential of 100 *μ*V (recorded from the right abductor pollicis brevis) in at least 50% of a series of ten single pulses applied to the left motor cortex. MT was assessed for both experimental sessions separately. Mean pulse intensity for all subjects was 47.4% (ranging from 32% to 60%) of the maximal stimulator intensity.

### 2.4. Statistical Analysis

Regarding the discrimination data, psychometric functions were calculated for each subject and each session. Logistic functions were fitted using R package “modelfree,” representing the probability of the response “comparison was longer than standard” depending on the comparison duration. Guessing and lapsing rates were set to .001. In order to quantify mean accuracy and precision of temporal judgments, we extracted the point of subjective equality (PSE) and the difference limen (DL). The data of four participants had to be excluded from this analysis, because the PSE was outside the range of the tested comparison durations (all were perceived as longer than the standard).

Regarding the reproduction data, we calculated the ratios between reproduced and standard durations. Values exceeding three times the standard deviation of the respective participant in the respective condition (0.4% of trials) were discarded as outliers. Median reproductions were calculated for each standard duration, and power functions of the form

(1) $$\begin{array}{c} f\left(\;x\;\right)=k\times x^{e} \end{array}$$

were fitted for each subject and each session. The constant *k* determines the scale unit, *x* denotes the standard, and *e* is the power exponent. To quantify individual reproduction accuracy, we extracted the power exponent *e* and the median of reproduction/standard ratios (aggregated for all standards). Individual precision was quantified by the variability of reproductions, defined by the interquartile range of reproduction/standard ratios. Statistical analysis was performed using *t*-tests for paired samples (two-tailed) and correlation analyses.

## 3. Results

**Figure 3:**
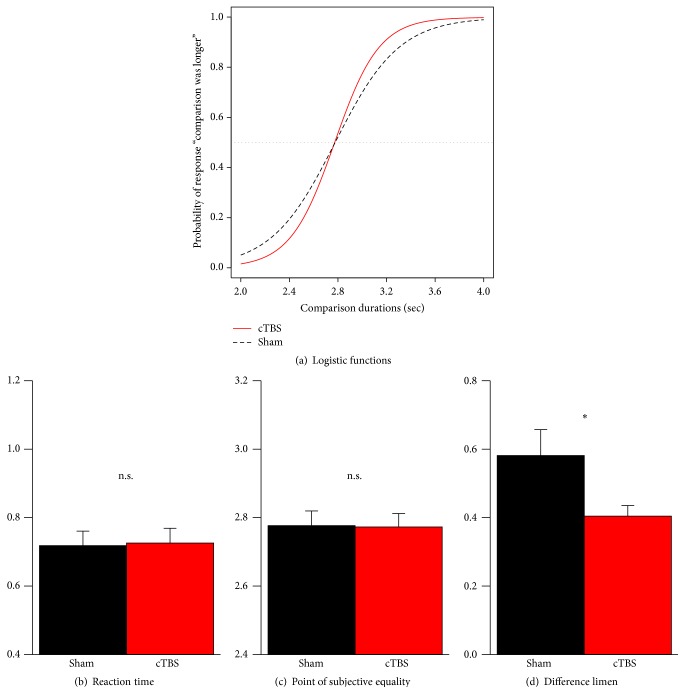
(a) Averaged logistic functions according to the discrimination data. No differences between cTBS (red bars) and sham stimulation (black bars) were found for reaction times (b) and the point of subjective equality (c). In contrast, reduction of the difference limen (d) indicates increased precision and thus a higher judgment certainty after cTBS. Error bars show standard errors of the mean across subjects.

Results for the discrimination task are depicted in Figure 3. Inhibition of the right PPC significantly reduced the DL, indicating increased precision of temporal judgments (*t*_19_ = −2.4, *p* = .03), but had no influence on the PSE, indicating stable mean accuracy (*t*_19_ = −0.1, *p* > .5). These results validate the experimental manipulation used here. We were able to manipulate judgment certainty for temporal intervals independent of a shift of the psychometric function, which would denote a bias towards either “shorter” or “longer” judgments and thus a change in mean accuracy. No differences in overall reaction times were observed between TMS and sham stimulation (*t*_19_ = 0.2, *p* > .5).

**Figure 4:**
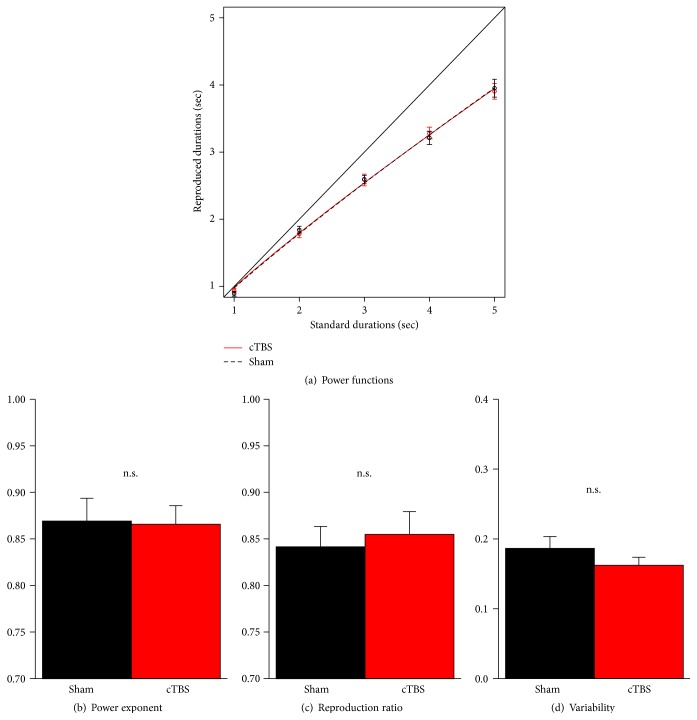
(a) Averaged power functions according to the reproduction data. No differences between cTBS (red bars) and sham stimulation (black bars) were found for the power exponent (b), the ratio between reproduced and standard durations, (c) and response variability (d). Error bars show standard errors of the mean across subjects.

Reproduction performance is depicted in Figure 4. PPC inhibition had no effect on accuracy, reflected neither by the power exponent (*t*_23_ = −0.1, *p* > .5) nor by the ratio between reproduced and standard durations (*t*_23_ = 0.8, *p* = .42). Furthermore, cTBS did not influence precision, that is, the variability of responses in the reproduction task (*t*_23_ = −0.6, *p* > .5). Analysis of the coefficient of variation (CV) revealed the same results (*t*_23_ = −1.5, *p* = .14). Average CV was .17 after sham stimulation and .15 after cTBS.

Another test for our initial hypothesis that increased precision in discrimination tasks would coincide with an attenuation of reproduction errors is provided by correlation analyses. According to the hypothesis, individuals showing the highest TMS-induced precision increase in time discrimination should concurrently show a more pronounced attenuation of reproduction errors. However, this prediction was not confirmed by the data. TMS-induced changes in discrimination precision did correlate neither with respective changes in the power exponent for reproduction (*t*_18_ = 0.6, *p* > .5, *r* = .14) nor with changes in reproduction errors (*t*_18_ = −0.8, *p* = .45, *r* = −.18).

Given that time discrimination and time reproduction tasks were differentially affected by inhibition of the right PPC, it might be speculated whether both tasks are based on different processes. Therefore, we analyzed the correlation between accuracy and precision within the reproduction task, asking whether TMS-induced changes in response variability are related to an attenuation of underreproduction errors. No significant correlation was found (*t*_22_ = −0.8, *p* = .4, *r* = −.18).

## 4. Discussion

We proposed that systematic negative errors in time reproduction tasks are caused by a general judgment bias towards earlier responses, instead of reflecting a genuine misperception of temporal intervals [3, 4]. Here we tested whether different levels of uncertainty regarding temporal judgments can influence such a bias. Temporal certainty was manipulated by application of continuous theta-burst stimulation (cTBS) over the intraparietal sulcus (IPS) in the right posterior parietal cortex (PPC), an area involved in timing judgments [7, 8, 11–13, 17, 18].

Changes in certainty were reflected in increased precision in a time discrimination task after application of TMS, while the mean accuracy of discriminative judgments remained stable. In spite of this successful manipulation check, TMS had no effect on the performance in a time reproduction task. Both the precision and the accuracy of reproduced time intervals, quantified by response variability and mean errors, respectively, were not affected by the conditions. Accordingly, we can conclude that the certainty of temporal judgments is unrelated to errors in time reproduction. In line with this interpretation, we found no correlation between increased precision in the discrimination task and attenuated errors in the reproduction task, which would be expected if judgment certainty was a principal cause for the underreproduction of temporal intervals.

There are several reasons which potentially can explain the judgment bias towards earlier responses in reproduction tasks. First, uncertainty regarding temporal judgments can reduce the criterion level for the termination of the reproduction interval (as illustrated in Figure 1). Second, adaptation to previously presented stimuli (adaptation-level effects, [19]) can cause a systematic shift towards shorter durations, because the presentation of each interval is necessarily preceded by the immediate experience of shorter intervals [3]. Third, the awareness that we cannot go back in time and that the correct moment will be irretrievably missed when waiting too long can induce the urge to terminate the reproduction interval rather too early than too late. The first of these potential explanations is ruled out by the present study. The underreproduction of temporal intervals is independent of judgment uncertainty. The other possibilities need to be considered in future studies. For example, the third point might be addressed by analyzing the correlation between parameters of individual risk tolerance and individual underreproduction bias.

The results of the present study reveal a difference between the psychophysical methods of time discrimination and time reproduction and suggest that the two tasks are based on different neuronal mechanisms. Specifically, the reproduction performance seems to be independent of the certainty regarding temporal judgments, which we were able to manipulate by applying cTBS over the IPS within the right PPC, an area which is well known for its involvement in the processing of temporal intervals [8, 11, 13, 18]. However, the role of the PPC for the perception of time has mainly been investigated using discrimination tasks, while the application of other psychophysical methods in this regard is rather scarce. In recent years, it was increasingly acknowledged that many findings in time perception research depend on the nature of the psychophysical method used [3, 20, 21]. For example, patients suffering from attention-deficit/hyperactivity disorder and autism spectrum disorders show impairments in the reproduction of temporal intervals [22–24], but they perform equal to healthy controls when measured with other methods [25–27]. Our results are in line with this argument, as they demonstrate that time reproduction and time discrimination tasks are differentially affected by a transient inhibition of the right PPC.

Several previous studies have investigated the role of parietal areas for time discrimination and reproduction (e.g., [8, 28–30]). For example, Hayashi et al. [29] reported that application of cTBS over the right intraparietal cortex affected performance in a time reproduction but not in a time discrimination task. Harrington et al. [28] even reported a decreased precision in time discrimination in a patient group with right-hemispheric lesions. These findings seem to contrast with our result of increased precision in time discrimination and unaltered reproduction performance after inhibition of the right PPC.

A possible explanation for these different results is provided by the different range of intervals used [7]. In the present study, we implemented suprasecond intervals, while subsecond intervals were tested in Harrington et al. ([28]; 300–600 ms) and Hayashi et al. ([29]; 450–900 ms). There is much evidence for the existence of different neuronal mechanisms underlying the processing of subsecond and suprasecond intervals [7, 31, 32], and Lewis and Miall [31] found parietal areas to be especially recruited during discrimination of suprasecond intervals (3 s). The present study extends previous research by showing that cTBS-induced inhibition of the right PPC affects the precision for time discrimination in the suprasecond range, while the reproduction of suprasecond durations is unaltered. In this regard, it is interesting to note that Oliveri et al. [30] reported no effect of right PPC inhibition on the reproduction of relatively long intervals (1.6 to 2.4 seconds), while performance was affected when subjects were asked to stop the interval after half of the standard duration had elapsed (i.e., 800–1200 ms).

In accordance with a previous study, we found an effect of TMS on the precision in a time discrimination task [8]. However, our results deviate from this study in the fact that we show a precision increase, while Bueti et al. reported a precision decrease. This difference can be explained by the different TMS protocols used. Instead of online stimulation during the experimental task, we applied continuous theta-burst stimulation (cTBS), an offline protocol generally known for its transient inhibitory effects [14–16]. Bueti et al. applied a train of seven pulses at a frequency of 12 Hz, a protocol commonly associated with excitatory effects [33]. Thus, together with Bueti et al. [8], our results demonstrate that TMS over the right PPC not only can disrupt but also improve the precision in time discrimination tasks. This observation illuminates the role the right PPC plays for time perception. Parietal areas are known as key structures for the integration of sensory inputs from different modalities [9, 18, 34–39]. Interactions between the perceptions of temporal, spatial, and numerical stimuli are mediated by neuronal structures within the PPC [18, 29, 30, 40]. Thus, PPC inhibition might suppress these multisensory interferences, leaving more resources for the processing of pure temporal stimuli. On the contrary, PPC excitation might stimulate the processing of combined stimuli, thereby reducing the precision for pure time judgments.

## 5. Conclusions

The psychophysical method of time reproduction has consistently revealed a systematic underreproduction of temporal intervals, that is, a judgment bias towards earlier rather than later responses [5]. The present study shows that these negative errors are not caused by temporal uncertainty. Increased certainty regarding temporal judgments had no effect on the magnitude of time underreproduction.

## References

[1] Fechner G. T. (1860). *Elemente der Psychophysik (Erster Theil)*.

[2] Gescheider G. A. (1997). *Psychophysics: The Fundamentals*.

[3] Riemer M. (2015). Psychophysics and the anisotropy of time. *Consciousness and Cognition*.

[4] Riemer M., Trojan J., Kleinböhl D., Hölzl R. (2012). A ‘view from nowhen’ on time perception experiments. *Journal of Experimental Psychology: Human Perception and Performance*.

[5] Eisler H. (1976). Experiments on subjective duration 1868–1975: a collection of power function exponents. *Psychological Bulletin*.

[6] Wackermann J., Ehm W. (2006). The dual klepsydra model of internal time representation and time reproduction. *Journal of Theoretical Biology*.

[7] Wiener M. (2014). Transcranial magnetic stimulation studies of human time perception: a primer. *Timing & Time Perception*.

[8] Bueti D., Bahrami B., Walsh V. (2008). Sensory and association cortex in time perception. *Journal of Cognitive Neuroscience*.

[9] Coull J. T., Nobre A. C. (1998). Where and when to pay attention: The neural systems for directing attention to spatial locations and to time intervals as revealed by both PET and fMRI. *Journal of Neuroscience*.

[10] Leon M. I., Shadlen M. N. (2003). Representation of time by neurons in the posterior parietal cortex of the macaque. *Neuron*.

[11] Maquet P., Lejeune H., Pouthas V. (1996). Brain activation induced by estimation of duration: a PET study. *NeuroImage*.

[12] Onoe H., Komori M., Onoe K., Takechi H., Tsukada H., Watanabe Y. (2001). Cortical networks recruited for time perception: a monkey positron emission tomography (PET) study. *NeuroImage*.

[13] Rao S. M., Mayer A. R., Harrington D. L. (2001). The evolution of brain activation during temporal processing. *Nature Neuroscience*.

[14] Chaves S., Vannini P., Jann K. (2012). The link between visual exploration and neuronal activity: A multi-modal study combining eye tracking, functional magnetic resonance imaging and transcranial magnetic stimulation. *NeuroImage*.

[15] Huang Y.-Z., Edwards M. J., Rounis E., Bhatia K. P., Rothwell J. C. (2005). Theta burst stimulation of the human motor cortex. *Neuron*.

[16] Nyffeler T., Cazzoli D., Wurtz P. (2008). Neglect-like visual exploration behaviour after theta burst transcranial magnetic stimulation of the right posterior parietal cortex. *European Journal of Neuroscience*.

[17] Battelli L., Pascual-Leone A., Cavanagh P. (2007). The ‘when’ pathway of the right parietal lobe. *Trends in Cognitive Sciences*.

[18] Bueti D., Walsh V. (2009). The parietal cortex and the representation of time, space, number and other magnitudes. *Philosophical Transactions of the Royal Society B: Biological Sciences*.

[19] Helson H. (1964). *Adaptation-Level Theory*.

[20] Droit-Volet S., Wearden J. H., Zelanti P. S. (2015). Cognitive abilities required in time judgment depending on the temporal tasks used: a comparison of children and adults. *The Quarterly Journal of Experimental Psychology (Hove)*.

[21] Gil S., Droit-Volet S. (2011). ‘Time flies in the presence of angry faces’... depending on the temporal task used!. *Acta Psychologica*.

[22] Barkley R. A., Koplowitz S., Anderson T., Mcmurray M. B. (1997). Sense of time in children with ADHD: effects of duration, distraction, and stimulant medication. *Journal of the International Neuropsychological Society*.

[23] Martin J. S., Poirier M., Bowler D. M. (2010). Brief report: impaired temporal reproduction performance in adults with autism spectrum disorder. *Journal of Autism and Developmental Disorders*.

[24] Szelag E., Kowalska J., Galkowski T., Pöppel E. (2004). Temporal processing deficits in high-functioning children with autism. *British Journal of Psychology*.

[25] Barkley R. A., Murphy K. R., Bush T. (2001). Time perception and reproduction in young adults with attention deficit hyperactivity disorder. *Neuropsychology*.

[26] Bauermeister J. J., Barkley R. A., Martinez J. V. (2005). Time estimation and performance on reproduction tasks in subtypes of children with attention deficit hyperactivity disorder. *Journal of Clinical Child & Adolescent Psychology*.

[27] Gil S., Chambres P., Hyvert C., Fanget M., Droit-Volet S. (2012). Children with Autism Spectrum Disorders have ‘the working raw material’ for time perception. *PLoS ONE*.

[28] Harrington D. L., Haaland K. Y., Knight R. T. (1998). Cortical networks underlying mechanisms of time perception. *Journal of Neuroscience*.

[29] Hayashi M. J., Kanai R., Tanabe H. C. (2013). Interaction of numerosity and time in prefrontal and parietal cortex. *Journal of Neuroscience*.

[30] Oliveri M., Koch G., Salerno S., Torriero S., Gerfo E. L., Caltagirone C. (2009). Representation of time intervals in the right posterior parietal cortex: implications for a mental time line. *NeuroImage*.

[31] Lewis P. A., Miall R. C. (2003). Brain activation patterns during measurement of sub- and supra-second intervals. *Neuropsychologia*.

[32] Wiener M., Turkeltaub P., Coslett H. B. (2010). The image of time: a voxel-wise meta-analysis. *NeuroImage*.

[33] Hallett M. (2007). Transcranial magnetic stimulation: a primer. *Neuron*.

[34] Basso G., Nichelli P., Frassinetti F., Di Pellegrino G. (1996). Time perception in a neglected space. *NeuroReport*.

[35] Burr D. C., Ross J., Binda P., Morrone M. C. (2010). Saccades compress space, time and number. *Trends in Cognitive Sciences*.

[36] Hubbard E. M., Piazza M., Pinel P., Dehaene S. (2005). Interactions between number and space in parietal cortex. *Nature Reviews Neuroscience*.

[37] Magnani B., Oliveri M., Mangano G. R., Frassinetti F. (2010). The role of posterior parietal cortex in spatial representation of time: a TMS study. *Behavioural Neurology*.

[38] Oliveri M., Rausei V., Koch G., Torriero S., Turriziani P., Caltagirone C. (2004). Overestimation of numerical distances in the left side of space. *Neurology*.

[39] Walsh V. (2003). A theory of magnitude: common cortical metrics of time, space and quantity. *Trends in Cognitive Sciences*.

[40] Göbel S. M., Calabria M., Farnè A., Rossetti Y. (2006). Parietal rTMS distorts the mental number line: simulating ‘spatial’ neglect in healthy subjects. *Neuropsychologia*.

